# Innovative Z-Scheme Heterojunction Photocatalyst ZnBiGdO_4_/SnS_2_ for Photocatalytic Degradation of Tinidazole Under Visible Light Irradiation

**DOI:** 10.3390/ijms26178366

**Published:** 2025-08-28

**Authors:** Jingfei Luan, Boyang Liu, Liang Hao, Wenchen Han, Anan Liu

**Affiliations:** 1 School of Physics, Changchun Normal University, Changchun 130032, China; boyangliu152@outlook.com (B.L.); 19845486007@139.com (L.H.); han18635869581@outlook.com (W.H.); ananliu2001@outlook.com (A.L.); 2 State Key Laboratory of Pollution Control and Resource Reuse, School of the Environment, Nanjing University, Nanjing 210093, China

**Keywords:** heterojunction photocatalyst ZnBiGdO_4_/SnS_2_, Z-type mechanism, tinidazole, photocatalytic degradation efficiency, degradation pathway, visible light irradiation

## Abstract

A high-performance Z-scheme heterojunction photocatalytic compound, ZnBiGdO_4_/SnS_2_ (ZS), was prepared for the first time using a microwave-assisted solvothermal method. ZS significantly improved the separation efficiency of photoinduced carriers and effectively broadened the response range to visible light through the unique mechanism of the Z-type heterojunction. Therefore, ZS exhibited an excellent photocatalytic performance during the degradation process of tinidazole (TNZ). Specifically, the removal rate of TNZ by ZS reached 99.63%, and the removal rate of total organic carbon (TOC) reached 98.37% with ZS as catalyst under visible light irradiation (VLIN). Compared to other photocatalysts, the photocatalytic performance of ZS was significantly better than that of ZnBiGdO_4_, SnS_2_, or N-doped TiO_2_ (N-T). The removal rate of TNZ by ZS was 1.12 times, 1.26 times, or 2.41 times higher than that by ZnBiGdO_4_, SnS_2_, or N-T, respectively. The mineralization efficiency of TNZ for TOC with ZS as a catalyst was 1.15 times, 1.28 times, or 2.57 times higher than that with ZnBiGdO_4_, SnS_2_, or N-T as a catalyst, respectively. Free radical scavenging experiments and the electron paramagnetic resonance experiments confirmed that ZS could generate multiple reactive species such as hydroxyl radicals (•OH), superoxide anions (•O_2_^−^), and photoinduced holes (h^+^) during the photocatalytic degradation process of TNZ. The photocatalytic degradation performance of ZS on TNZ under VLIN was evaluated, concurrently, the reliability, reproducibility, and stability of ZS were verified by five cycle experiments. This study explored the degradation mechanism and degradation pathway of TNZ with ZS as a catalyst under VLIN. This study not only provides new ideas for the design and preparation of Z-type heterojunction photocatalysts but also lays an important foundation for the development of efficient environmental remediation technologies for TNZ pollution.

## 1. Introduction

In recent decades, tinidazole (TNZ, C_8_H_13_N_3_O_4_S), a second-generation nitroimidazole derivative, had emerged as a clinically important antianaerobic-microbacterial drug. TNZ had been primarily used for the treatment and prevention of infections caused by anaerobic bacteria or certain protozoa, such as oral infection, gynecological inflammation, and intestinal amoebiasis [1]. Due to the favorable efficacy and tolerability of TNZ, along with it having fewer side effects, TNZ had been widely used in North America, Europe, and the Asia-Pacific region. Consequently, large amounts of TNZ had entered aquatic environments through pharmacy and hospital wastewater discharge and human metabolic excretion. Furthermore, due to the incomplete metabolism and difficult biodegradation ability of TNZ in water systems, TNZ could persist in the ecosystem for a long time. This had led to severe TNZ water pollution problems; thereby, a serious threat was posed to the ecosystem. Therefore, removing TNZ from wastewater was of particular importance [2].

Previously, various methods had been applied to eliminate TNZ from wastewater, including precipitation, electrochemical treatment, ion exchange, reverse osmosis, and green membrane filtration. However, these methods possessed imperfections such as high energy consumption, prohibitive cost, and secondary pollution [3]. Therefore, they could not be applied in practical water pollution control engineering. Hence, developing green technology and efficient technology for removing TNZ carried significant scientific importance and practical urgency.

Photocatalytic advanced oxidation technology emerged as an environmental purification technology. Photocatalytic advanced oxidation technology had attracted increasing attention from researchers owing to its advantages such as low cost, high efficiency, and low energy consumption. However, traditional metal oxides and metal sulfides photocatalysts such as ZnO, TiO_2_, CdS, and MoS_2_ faced many deficiencies in practical applications. For example, the above photocatalysts had wide band gaps, high recombination rates of photoinduced electrons and photoinduced holes, and a limited number of active sites [4]. In response to the above challenges, the development of novel photocatalysts with efficient visible light response ability, high carrier separation efficiency, and a large specific surface area became a core direction in the current field of photocatalysis.

The AB_2_O_4_-type oxide photocatalyst possessed great potential for the efficient degradation of various organic pollutants owing to its enhanced visible light responsiveness, excellent thermal stability, outstanding chemical stability, large specific surface area, and rich active sites. For example, Derkaoui K et al. prepared the photocatalyst MnFe_2_O_4_ by a coprecipitation method, which achieved a degradation rate of 68.51% for Rhodamine B within 90 min under visible light irradiation (VLIN) [5]. Muthukrishnaraj A et al. prepared CuBi_2_O_4_ with a one-step hydrothermal method; as a result, the removal rate of 40% for degrading methylene blue was achieved under VLIN within 100 min [6]. In addition, Krishnakumar et al. synthesized Zn-doped TiO_2_ by the sol–gel method; under VLIN, a significantly higher degradation rate of reactive yellow 145 was exhibited compared with pure TiO_2_ [7]. Meanwhile, Zhu et al. synthesized Bi-doped TiO_2_ using the sol–gel method, and the photocatalytic degradation efficiency of methyl orange by the Bi-doped TiO_2_ photocatalyst was significantly improved compared to that by pure TiO_2_ [8]. In addition, Tang et al. evaluated the performance of a Gd-doped TiO_2_ photocatalyst, and the experimental results indicated that Gd-doped TiO_2_ exhibited drastically enhanced photocatalytic activity for degrading methylene blue compared with pure TiO_2_ under VLIN [9]. Building upon these foundational findings, this study rationally designed and fabricated ZnBiGdO_4_, a newly developed visible-light-driven photocatalyst with enhanced charge separation efficiency.

However, single photocatalysts faced numerous challenges, such as the high recombination rate of photogenerated charge carriers, in practical applications. Previous research findings had revealed that constructing a heterojunction could enhance the photocatalytic performance of the photocatalysts. The internal electric field that was generated between two components of the heterojunction could efficiently achieve the spatial separation of photogenerated carriers. Concurrently, a relatively high redox potential could be maintained. Consequently, more efficient reactive radicals such as hydroxyl radicals, superoxide anions, and photoinduced holes with strong oxidative capacity were generated. Zhao et al. successfully synthesized the g-C_3_N_4_/BiNbO_4_ composite photocatalyst by a facile ultrasound-assisted impregnation route; as a result, g-C_3_N_4_/BiNbO_4_ exhibited an excellent catalytic performance. After VLIN of 150 min, the removal rate of TNZ by g-C_3_N_4_/BiNbO_4_ reached 90%, which was significantly superior than that by the single-component g-C_3_N_4_ (approximately 70%) or that by BiNbO_4_ (67%) [10].The direct Z-scheme g-C_3_N_4_/TiO_2_ heterojunction photocatalyst that was prepared by Jo et al. via a wet impregnation method was used as catalyst, and as a result, a pollutant removal rate of 90.8% for degrading isoniazid was achieved within 240 min under VLIN. The removal rate of isoniazid by the g-C_3_N_4_/TiO_2_ heterojunction photocatalyst was significantly higher than that by TiO_2_ (73.3%) or that by g-C_3_N_4_ (13.5%) [11].

Currently, most available AB_2_O_4_-type oxide heterojunctions were composed of one AB_2_O_4_-type spinel oxide and another oxide or polymer semiconductor such as g-C_3_N_4_. For example, Qu et al. synthesized the Z-scheme BiVO_4_/NiCo_2_O_4_ heterojunction photocatalyst by decorating ultrathin nickel-cobalt (NiCo_2_O_4_) spinel nanosheets onto BiVO_4_ [12]. Renukadevi, S. et al. constructed a novel ZnFe_2_O_4_/g-C_3_N_4_ heterojunction photocatalyst using a microwave irradiation method [13]. Liang et al. fabricated a NiAl_2_O_4_/g-C_3_N_4_ heterojunction photocatalyst by synthetizing g-C_3_N_4_ onto spinel oxide NiAl_2_O_4_ [14].

Nevertheless, the studies on the construction of heterojunctions, which were based on spinel-type oxides and sulfides, were relatively restricted. SnS_2_ was a direct band gap semiconductor catalyst with excellent photocatalytic performance. SnS_2_ was commonly used to prepare composite photocatalysts. Zhang et al. synthesized a high-performance visible light-driven SnS_2_/SnO_2_ nanometer heterojunction photocatalyst via a one-step hydrothermal method, and SnS_2_/SnO_2_ exhibited high catalytic activity; simultaneously, the photocatalytic efficiency of SnS_2_/SnO_2_ was superior in contrast to that of a pure SnS_2_ nanosheet [15]. Zhou et al. constructed a flower-like ZnO/SnS_2_ heterojunction, and as a result, the ZnO/SnS_2_ heterojunction exhibited a higher photocatalytic efficiency in degrading organic dyes compared with pure SnS_2_; concurrently, the removal rate of the organic dyes by ZnO/SnS_2_ was approximately 2.7 times than that by SnS_2_ [16]. Based on the above findings, our study designed and synthesized a direct Z-scheme ZnBiGdO_4_/SnS_2_ (ZS) heterojunction photocatalyst. In this study, the N-doped TiO_2_ (N-T) was prepared as a contrapositive photocatalyst to evaluate the photocatalytic activity of SnS_2_, ZnBiGdO_4_, and ZS.

As a novel photocatalytic compound, ZS achieved a good separation efficiency of photoinduced charge carriers by means of the unique structural design and the drastically extended visible light response range for ZS. This study presents the supereminent efficiency and innovation of the ZS catalyst during the degradation of TNZ; ultimately, the further practical application of photocatalytic technology in the field of environmental remediation is promoted.

## 2. Results and Discussion

### 2.1. Characterization of Photocatalysts

The phase compositions of the fabricated ZnBiGdO_4_ sample, SnS_2_ sample, and ZS sample were analyzed by the X-ray diffraction (XRD) experiment. Figure S3a shows the XRD pattern and Pawley refinement result for ZnBiGdO_4_. According to Figure S3a, the experimental result showed that the fabricated ZnBiGdO_4_ possessed obvious sharp diffraction peaks. The Pawley method of Materials Studio software (2023 version) was used to refine the XRD experimental data of ZnBiGdO_4_, and many parameters were optimized. It can be found from the refinement result in Figure S3a that the Rp factor of ZnBiGdO_4_ was 4.49%; concurrently, ZnBiGdO_4_ belonged to the body-centered tetragonal system with spinel structure, and space group was I41/AMD; moreover, the cell parameters for ZnBiGdO_4_ were a = b = 14.622367 Å, c = 9.513909 Å, and ultimately, the volume of monocelled ZnBiGdO_4_ was 2340 (Å)^3^. The interplanar spacing of the crystal plane with Miller indices (103) for ZnBiGdO_4_ was 0.310 nm. The relevant atomic coordinates and structural parameters of ZnBiGdO_4_ are listed in Table S1. The final refinement results of ZnBiGdO_4_ showed that the experimentally observed intensity was in good agreement with the calculated intensity in the tetragonal spinel structure. Sharp diffraction peaks were present in the pattern of ZnBiGdO_4_, and the diffraction peaks of impurities were not observed. The above results indicated that the synthesized ZnBiGdO_4_ sample had high crystallinity; simultaneously, the impurities or secondary phases did not exist within ZnBiGdO_4_. The above results also laid a good foundation for the successful preparation of the pure ZS sample with high catalytic performance. The structure of ZnBiGdO_4_ is shown in Figure S3b. In the structure of ZnBiGdO_4_, there were two bonds that connected with Zn; meanwhile, above two bonds were Zn-O bonds with a bond length of 2.24936 Å. There were three bonds which connected with bismuth, concurrently, above three bonds were Bi-O bonds; nevertheless, the bond lengths were different. Among them, one Bi-O(1) bond possessed a bond length of 2.39513 Å, and two Bi-O(3) bonds had a bond length of 2.56324 Å. According to the central bismuth atom, the O(3)-Bi-O(3) bond angle was 92.133°; simultaneously, the O(1)-Bi-O(3) bond angle was 87.382°. When the M-O-M angle was closer to 180°, the mobility of photogenerated carriers was stronger, and as a result, a better catalytic performance could be achieved. Previous studies have demonstrated that the photocatalytic activity was enhanced when the M-O-M bond angle approached 180°. For example, in YbDyBiNbO_7_, the M-O-M (M = Bi^3+^ and Nb^5+^) bond angle of 133.273° approached the ideal 180°; accordingly, the photocatalytic activity of the catalyst was significantly enhanced. Based on the central oxygen atom, the Bi-O(3)-Bi bond angle was 137.362°, which enabled the photocatalyst to have a better photocatalytic performance [17,18,19].

The Pawley refinement of the XRD experimental data of SnS_2_ was performed using Materials Studio software, and many parameters were optimized. The atomic coordinates and structural parameters of S and Sn for SnS_2_ are shown in Table S2. Figure S4a indicates the XRD pattern and Pawley refinement result of SnS_2_. It can be seen from Figure S4a that the Rp factor of SnS_2_ is 6.71%. According to Figure S4a, the experimental result and the calculated result for SnS_2_ are in good agreement; meanwhile, the impurity peaks did not exist within SnS_2_ [20]. The space group of SnS_2_ was P-3M1 (164); simultaneously, SnS_2_ was found to belong to the hexagonal system, with a layered structure, in rapid sequence, and the cell parameters of SnS_2_ were found as the following: a = b = 3.6486 Å, c = 5.8992 Å. Finally, the volume of SnS_2_ was 67.43 (Å)^3^ and the structure type of SnS_2_ was CdI_2_ [21]. The structure of SnS_2_ is shown in Figure S4b. It can be discovered from Figure S4b that the S-Sn bond length for SnS_2_ was 2.59492 Å, and the bond angles for SnS_2_ were all 89.005°. The interplanar spacing that corresponds to the crystal plane index (001) for SnS_2_ was 0.589 nm. The prepared SnS_2_ matched well with the standard card (PDF#23-0677), indicating that SnS_2_ did not possess other impurities and the prepared SnS_2_ was a single phase with very high purity.

**Figure 1:**
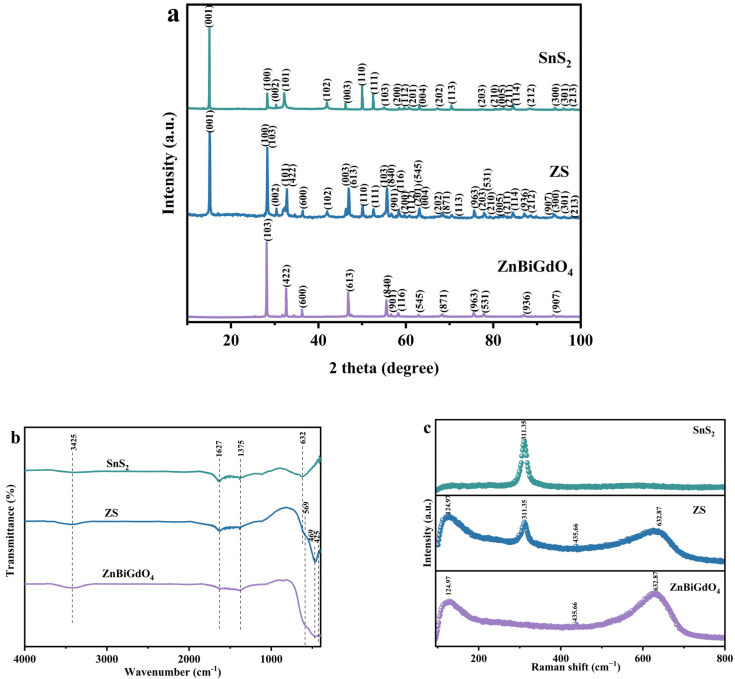
(**a**) XRD patterns, (**b**) FTIR spectrograms, and (**c**) Raman plots of ZnBiGdO_4_, SnS_2_, and ZS.

Figure 1a shows the XRD images of SnS_2_, ZnBiGdO_4_, and ZS. In the XRD image of ZS, all diffraction peaks and crystal planes were derived from the single phase of ZnBiGdO_4_ or SnS_2_. Such results preliminarily confirmed the successful synthesis of ZS.

In order to understand the interface characteristics, chemical bonds, and functional groups of the prepared photocatalysts, FTIR analysis was conducted. Figure 1b shows the FTIR spectra of ZnBiGdO_4_, SnS_2_, and ZS. It can be clearly observed from Figure 1b that the spectrum of ZS contained the vibration bands that belonged to ZnBiGdO_4_ and SnS_2_; thus, the above results provided further evidence for the successful synthesis of ZS. The vibration peak which located at 632 cm^−1^ confirmed the stretching vibration of the Sn-S bond [22]. The peak which located at 469 cm^−1^ or 425 cm^−1^ was the stretching vibration of the Zn-O bond or the Bi-O bond [23,24]. The vibration peaks observed at specific wavenumbers demonstrated the successful preparation of the SnS_2_ powder sample, ZnBiGdO_4_ powder sample, and ZS powder sample. In addition, the peak position which located at 3425 cm^−1^ corresponded to the stretching vibration of hydroxyl groups, which might originate from adsorbed water or water of crystallization. The absorption band which located at 1627 cm^−1^ indicated the presence of water, which corresponded to the bending vibration of the H–O–H structure. Furthermore, the peak which was observed around 1375 cm^−1^ was attributed to the stretching vibration mode of the C–H bond [25].

ZnBiGdO_4_, SnS_2_, and ZS were experimentally studied using a Raman spectrometer to understand the vibrational characteristic and microstructural characteristic of the above three photocatalysts. Figure 1c shows the Raman spectra of SnS_2_, ZnBiGdO_4_, and ZS. In the Raman spectrum of SnS_2_, a peak which located at 311.35 cm^−1^ could be observed; concurrently, above peak originated from the A1g vibration mode, and as a result, above Raman active vibration mode could be attributed to the out-of-plane stretching of the S atom [26]. In the Raman spectrum of ZnBiGdO_4_, a peak which located at 632.87 cm^−1^, 124.97 cm^−1^ or 435.66 cm^−1^ could be observed. The peak which located at 124.97 cm^−1^ could be attributed to the stretching vibration of the Bi-O bond within ZnBiGdO_4_, and the symmetry was represented as B1 [27]. The peak which located at 632.87 cm^−1^ originated from another vibration mode of Bi-O. The peak which located at 435.66 cm^−1^ originated from the in-plane vibration of the Zn-O bond, and the symmetry was represented as E2 [28]. In the Raman spectra of ZS, the peak vibrations of ZnBiGdO_4_ and SnS_2_ could be clearly observed, and the peaks from other impurities were not observed. Combining with the experimental results of FTIR, above Raman spectra results further preliminarily confirmed the successful synthesis of the single-phase and the heterojunction catalysts that were prepared in this study.

**Figure 2:**
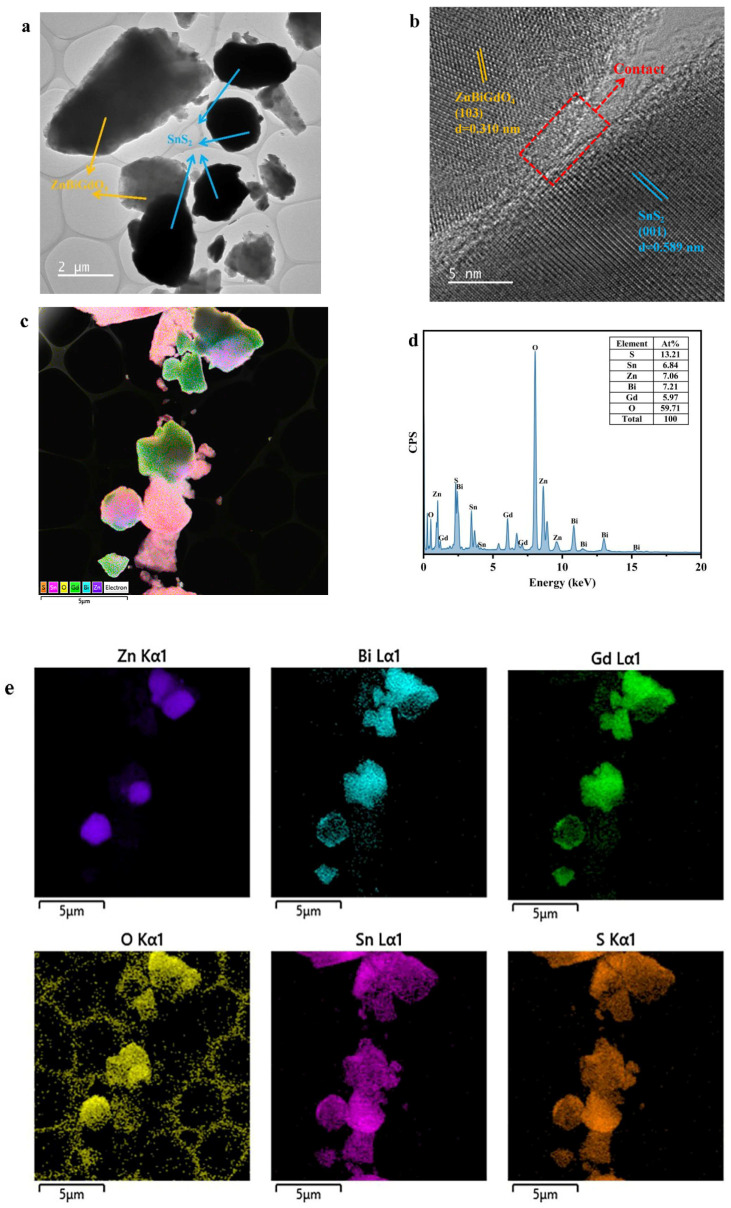
(**a**) TEM, (**b**) HRTEM, (**c**) layered EDS, (**d**) EDS, and (**e**) EDS elemental mapping images of ZS.

Transmission electron microscopy and energy-dispersive X-ray spectroscopy were used to analyze the microstructure and elemental composition of the ZS sample. Figure 2a shows the microstructure appearance of ZS. It could be found from Figure 2a that the larger size particles corresponded to ZnBiGdO_4_, while the smaller size particles which were in black color was SnS_2_. It could also be observed from Figure 2a that two single phases of SnS_2_ and ZnBiGdO_4_ were evenly distributed in space. Figure 2b indicates the high-resolution stripe imagery of ZS. Figure 2b revealed the interface information between SnS_2_ and ZnBiGdO_4_, as a result, the key role for promoting effective charge transfer between the various components was emphasized. Furthermore, according to Figure 2b, the lattice fringes with measured interplanar spacing of 0.589 nm or 0.310 nm was assigned to the (001) plane of hexagonal SnS_2_ or the (103) plane of ZnBiGdO_4_, respectively.

Figure 2c–e shows the EDS elemental mapping images of ZS. As shown in Figure 2c–e, the distribution of each element within the ZS was analyzed by elemental mapping method. It can be seen from Figure 2c–e that the Sn element, S element, Zn element, Bi element, Gd element, and O element were simultaneously distributed within the ZS sample; moreover, other elements were not detected. The above phenomenon indicated that there were two single phases of SnS_2_ and ZnBiGdO_4_ in ZS without other substances. The EDS spectrum shown in Figure 2d confirmed that the Sn element, S element, Zn element, Bi element, Gd element, and O element were distributed in the ZS photocatalyst according to the atomic ratio. Comprehensive analysis of the transmission electron microscope morphology and energy-dispersive X-ray spectroscopy illustrated that the prepared ZS sample possessed single-phase SnS_2_ and single-phase ZnBiGdO_4_, and the molar ratio of SnS_2_ and ZnBiGdO_4_ was approximately 1:1.

During the preparation process of ZS, SnS_2_ and ZnBiGdO_4_ did not undergo chemical changes for producing a new single-phase substance. Furthermore, combining with the results from X-ray diffraction (XRD), Fourier-transform infrared spectroscopy (FTIR), and Raman spectroscopy analyses, the above high-resolution stripe calculation results and EDS elemental mapping results confirmed both the feasibility of the synthetic approach and the successful preparation of the pure ZS sample.

**Figure 3:**
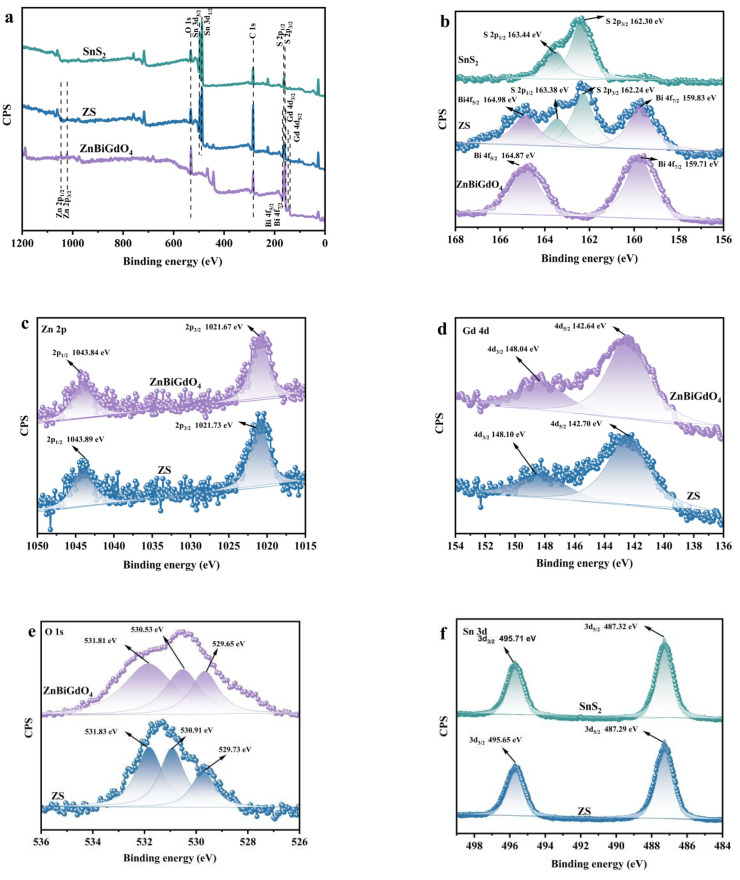
The XPS spectrum of synthesized ZnBiGdO_4_, SnS_2_, and ZS: (**a**) survey spectrum; (**b**–**f**) high-resolution spectra of Zn 2p, Bi 4f, Gd 4d, Sn 3d, S 2p, and O 1S, respectively.

In order to obtain information about the sample elements and chemical states, X-ray photoelectron spectroscopy experiment on the samples was performed. Figure 3 shows the XPS spectra of synthesized ZnBiGdO_4_, SnS_2_, or ZS. The survey spectrum of ZS shown in Figure 3a confirmed the presence of the S element, Sn element, Zn element, Bi element, Gd element, and O element, indicating that the above elements originated from SnS_2_ and ZnBiGdO_4_. In the Bi 4f orbital shown in Figure 3b, due to spin–orbit interaction, which could be divided into spin-up and spin-down, the Bi 4f orbital that belonged to ZnBiGdO_4_ could be split into two peaks that corresponded to Bi 4f_5/2_ and Bi 4f_7/2_; simultaneously, the peak position of Bi 4f_5/2_ appeared at 164.87 eV and the peak position of Bi 4f_7/2_ appeared at 159.71 eV [29]. At the same time, in ZS, two peak positions at 164.98 eV and at 159.83 eV showed a slight shift. For the Zn 2p orbital shown in Figure 3c, the binding energy position of the Zn 2p_1/2_ orbital in ZnBiGdO_4_ was at 1043.84 eV; concurrently, the binding energy position of the Zn 2p_3/2_ orbital was at 1021.67 eV [30]. In ZS, the binding energy position of the Zn 2p_1/2_ orbital was at 1043.89 eV; meanwhile, the binding energy position of the Zn 2p_3/2_ orbital was at 1021.73 eV. As to the Gd 4d orbital shown in Figure 3d, the peak position of Gd 4d_3/2_ in ZnBiGdO_4_ was at 148.04 eV; simultaneously, the peak position of Gd 4d_5/2_ was at 142.64 eV. In ZS, the peak position of Gd 4d_3/2_ was at 148.10 eV, in the meantime, the peak position of Gd 4d_5/2_ was at 142.70 eV. As to the O 1s orbital shown in Figure 3e, the peak positions of O 1s which belonged to ZnBiGdO_4_ were 531.81 eV, 530.53 eV, and 529.65 eV. In ZS, the peak positions of O 1s were at 531.83 eV, 530.91 eV, and 529.73 eV. The S 2p orbital shown in Figure 3b was split into S 2p_1/2_ and S 2p_3/2_ due to spin-orbit coupling; as a result, the peak position of S 2p_1/2_ was at 163.44 eV and the peak position of S 2p_3/2_ was at 162.30 eV. In ZS, the peak positions of S 2p_1/2_ and S 2p_3/2_ which were 163.38 eV and 162.24 eV produced a slight shift. For the Sn 3d orbital shown in Figure 3f, the binding energy of Sn 3d_3/2_ in SnS_2_ was 495.71 eV; concurrently, the binding energy of Sn 3d_5/2_ was 487.32 eV. In ZS, the binding energy of Sn 3d_3/2_ was 495.65 eV; simultaneously the binding energy of Sn 3d_5/2_ was 487.29 eV [24].

As to the Zn 2p orbital, Bi 4f orbital, Gd 4d orbital, and O 1s orbital, the binding energy positions in ZS possessed a slight positive shift compared to those in ZnBiGdO_4_. As to the S 2p orbital and Sn 3d orbital, the binding energy positions in ZS showed a slight negative shift compared with those in SnS_2_. The above phenomenon arose from the successful construction of a Z-scheme heterojunction, which induced electron density depletion in ZnBiGdO_4_ and concurrent electron accumulation in SnS_2_ [17]. At the same time, the aforementioned findings had shown the complex electronic interactions within the internal band structure of the ZS catalyst, which was essential for enhancing the functional properties of the ZS catalyst.

Notably, X-ray photoelectron spectroscopy (XPS) analysis of ZS, ZnBiGdO_4_, and SnS_2_ revealed that the secondary phases were not detectable. Comprehensive XPS results provided crucial evidence for developing a Z-scheme heterostructure in the ZS heterojunction. Combining with the above TEM results, the ZS catalyst further confirmed the strong chemical interaction between ZnBiGdO_4_ and SnS_2_. The above results complemented and reinforced the observations obtained from other characterization techniques such as X-ray diffraction, Fourier transform infrared spectroscopy, Raman spectroscopy, transmission electron microscopy, high-resolution transmission electron microscopy, and energy-dispersive X-ray spectroscopy.

**Figure 4:**
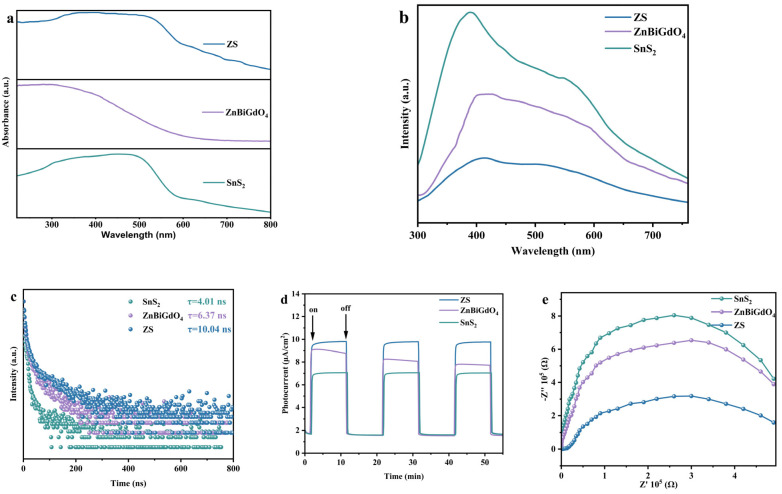
(**a**) UV–vis DRS; (**b**) PL spectra, (**c**) TRPL spectra, (**d**) PC curves, and (**e**) EIS plots of for ZnBiGdO_4_, SnS_2_, and ZS.

In order to investigate the optical response characteristic and band structure of the samples, the UV-Vis absorption spectra of SnS_2_, ZnBiGdO_4_, and ZS were measured. Figure 4a shows the absorption spectra of SnS_2_, ZnBiGdO_4_, and ZS. It can be observed from Figure 4a that SnS_2_, ZnBiGdO_4_, and ZS all exhibited significant absorption peaks in the range of 400 nm–700 nm, indicating that the samples were responsive to VLIN. *Eg* was determined by the intersection point between the *hυ* axis and the straight line of the linear extrapolation of the absorption edge. The curve is called the Kubelka–Munk formula [31].

(1) $$F_{KM}[R_{d}(h\nu )]=\frac{{\left[1-R_{d}(h\nu )\right]}^{2}}{2R_{d}(h\nu )}=\frac{\alpha h\nu }{S}$$

As to different transition mechanisms, the energy of incident photon and the *Eg* of the semiconductor compound possess the following simple relationships [32,33].

(2) $$\left(\alpha h\nu {)}^{n}=K(h\upsilon -Eg\right)$$

*α* is the absorption coefficient, *h* is Planck’s constant, *υ* is the frequency, *K* is constant, and *Eg* is the band gap. Within this equation, *n* determines the character of the transition in a semiconductor. *n* can be calculated by the following steps: (i) plotting *ln*(*αhυ*) versus *ln*(*hυ − Eg*) by assuming an approximate value of *Eg*, and (ii) deducing the value of *n* according to the slope in this graph. Based on above method, the value of *n* for the photocatalyzer could be ascertained. When *n* equaled 2 or 2/3, it represented a direct allowed transition and a direct forbidden transition, respectively; when n equaled 1/2 or 1/3, it represented an indirect allowed transition and an indirect forbidden transition, respectively [34,35].

Figure S5a displays the corresponding plot of (*αhν*)^1/2^ and *hν* for ZnBiGdO_4_. Figure S5b reveals the corresponding plot of (*αhν*)^2^ and *hν* for SnS_2_. Figure S5c shows the corresponding plot of (*αhν*)^2^ and *hν* for ZS. Figure S5 further details the estimated band gap energy values of SnS_2_, ZnBiGdO_4_, or ZS. SnS_2_ was a direct band gap with band gap width of 2.075 eV; correspondingly, ZnBiGdO_4_ was an indirect band gap with band gap width of 1.837 eV, and simultaneously, ZS was a direct band gap with band gap width of 1.821 eV. It can be concluded from Figure S5a,b that the band gap width of ZS was narrower compared with that of ZnBiGdO_4_ or SnS_2_. According to Figure 4a, the shape of the UV-Vis absorption spectrum for ZS appeared to be heavily influenced by that for SnS_2_, implying that the SnS_2_ phase that existed within ZS exhibited a greater influence. It can be found from Figure S5c that two band gaps appeared in ZS, indicating that ZS had two phases; accordingly, we could conclude that the band gap width of ZS was closely related with that of ZnBiGdO_4_ and SnS_2_. Two band gaps also implied that the electrons in ZnBiGdO_4_ and SnS_2_ exhibited different transition characteristics.

The above results indicate that under the same energy of VLIN, the ZS heterojunction catalyst could significantly reduce the energy barrier for electron transition, promoting more electrons for transition from the valence band to the conduction band. Thereby, the yield of the photoinduced electrons and the photoinduced holes could be increased; thus, more remarkable photocatalytic activity could be exhibited.

The photoluminescence spectra of SnS_2_, ZnBiGdO_4_, and ZS are shown in Figure 4b. In accordance with Figure 4b, the experimental results revealed that the photoluminescence intensity of the ZS sample was the lowest. At the emission peak located at the wavelength of 400 nm, the emission intensity of the ZS sample was lower than that of ZnBiGdO_4_ or SnS_2_. At the emission peak located at the wavelength of 570 nm, the emission intensity of the ZS sample was lower than that of ZnBiGdO_4_ or SnS_2_. It can be found from Figure 4b that the photoinduced electrons and the photoinduced holes generated in the ZS sample possessed the lowest recombination rate compared with those in the ZnBiGdO_4_ sample or in the SnS_2_ sample. Thus, ZS showed the strongest photocatalytic activity compared with ZnBiGdO_4_ or ZS. The above results further indicated that ZS showed the highest photocatalytic performance compared with ZnBiGdO_4_ or ZS [36,37]. The result was consistent with our previous analysis.

Time-resolved photoluminescence spectra are shown in Figure 4c. According to Figure 4c and the following formula, the fluorescence lifetime (*τ_ανe_*) of ZS can be calculated to be 10.04 ns. *τ_1_* and *τ_2_* are the corresponding parameters of the fluorescence lifetime. *A_1_* and *A_2_* are normalization analogue parameters.

(3) $$\tau_{ave}=(A_{1}\tau_{1}^{2}+A_{2}\tau_{2}^{2})/(A_{1}\tau_{1}+A_{2}\tau_{2})$$

Meanwhile, the fluorescence lifetime of SnS_2_ or ZnBiGdO_4_ was 4.01 ns or 6.37 ns, respectively. The above result implied that the photoinduced electrons and the photoinduced holes produced on the surface of the ZS particle were more difficult to recombine; thus, ZS achieved higher photocatalytic efficiency. The above results were consistent with the experimental results which were obtained in PL spectra, further demonstrating that the direct Z-scheme heterojunction ZS possessed the highest photocatalytic performance as compared to the single-component SnS_2_ and the single-component ZnBiGdO_4_.

In order to analyze the separation efficiency and diffusion efficiency of the photoinduced electrons and the photoinduced holes that were produced on the surface of the photocatalyst particle, the transient photocurrent response experiments were performed with SnS_2_, ZnBiGdO_4_, or ZS as the prepared membrane sample. The above experimental results are shown in Figure 4d. According to the experimental results derived from Figure 4d, the transient photocurrent intensity of the ZS sample was higher than that of the SnS_2_ sample or that of the ZnBiGdO_4_ sample. The above results demonstrated that with the irradiation of incident light, the ZS sample achieved the maximum utilization of light energy and the highest separation efficiency of the photogenerated electrons and the photogenerated holes.

ZS, SnS_2_, and ZnBiGdO_4_ exhibited different charge transfer characteristics according to the Nyquist plots of electrochemical impedance shown in Figure 4e. The semicircular diameter of the Nyquist plot for the ZS heterojunction catalyst was significantly smaller than that for the ZnBiGdO_4_ catalyst or that for the SnS_2_ catalyst, indicating that the ZS heterojunction catalyst possessed a lower charge transfer resistance. The above result suggested that the photogenerated carriers produced on the surface of the ZS particle had a higher migration efficiency compared with those of the ZnBiGdO_4_ particle or those of the SnS_2_ particle; as a result, above description was consistent with the excellent photocatalytic activity of the ZS heterojunction catalyst. The conclusion was mutually confirmed by the photocurrent measurement results and the PL spectral analysis results, further verifying that the ZS heterojunction catalyst had the superior performance as a highly efficient photocatalytic sample.

### 2.2. Photocatalytic Efficiency Measurement

#### 2.2.1. Photocatalytic Degradation of Tinidazole

**Figure 5:**
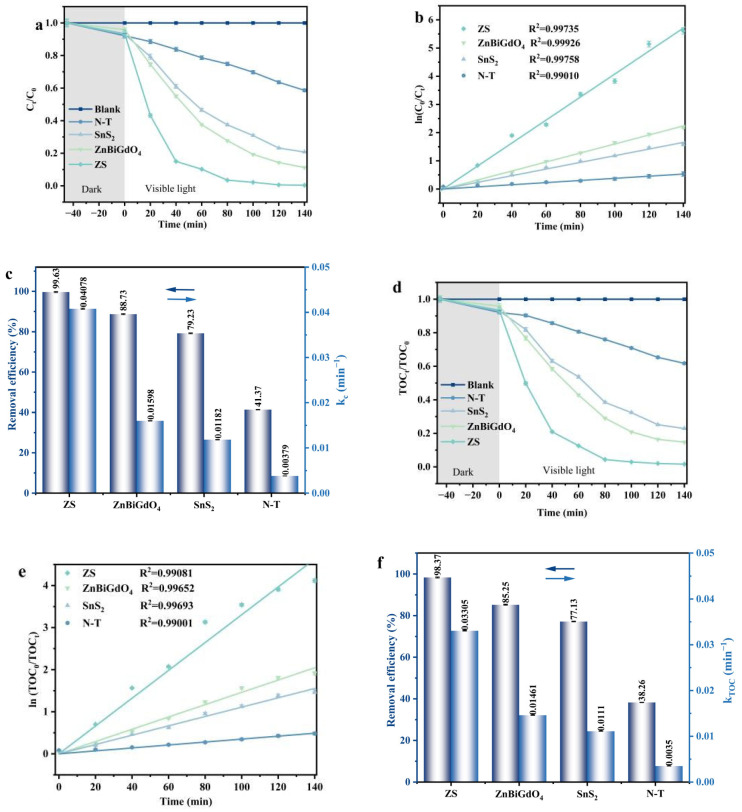
(**a**) Photodegradation, (**b**) kinetic curves, and (**c**) removal efficiencies and kinetic constants for TNZ; (**d**) mineralization, (**e**) kinetic curves, and (**f**) mineralization efficiencies and kinetic constants for TOC with ZS as photocatalyst under visible light irradiation.

Figure 5a shows the photo-driven catalytic performance of the photocatalyst for degrading TNZ under the condition of visible light irradiation. The solution was initially treated in the darkness for 45 min to achieve equilibrium between adsorption and desorption. The N-T sample has been repeatedly prepared and studied in the effluent treatment field because the N-T sample was a recognized visible-light responsive photocatalyst that could be widely used in the photocatalytic agent market. Figure S6 shows the XRD spectrum and the UV-Vis diffuse reflectance spectra of the N-T sample. The N-T sample was served as a contrapositive photocatalyst for evaluating the differences of the photocatalytic performance. It can be found from Figure S6 that the N-T sample showed the characteristic lattice planes of the anatase phase of TiO_2_ (JCPDS No. 21-1272) and the band gap energy value of the N-T sample was detected to be 2.92 eV. It can be found from Figure 5a that the removal rate of TNZ by ZS was significantly higher than that by ZnBiGdO_4_, SnS_2_, or N-T. By comparative experiments, it was found that the TNZ concentration did not change significantly without adding any catalyst. Above result indicated that the efficient removal of TNZ was due to the effect of the photocatalyst, rather than the photolysis reaction.

Figure 5b shows the plot of ln(*C*_0_/*C_t_*) versus time. It can be found from Figure 5b that the curve derived from the effect of ln(*C*_0_/*C_t_*) on VLIN time followed a first-order kinetic model. The first-order kinetic constant can be calculated using the following Formula.

(4) $$\ln(C_{0}/C_{t})=k_{C}t$$

where *C_t_* represents the TNZ concentrations at the intermediate state, *C*_0_ represents the TNZ concentration at the initial state, *k_C_* represents the kinetic constant, and t represents the irradiation time of visible light. Figure 5c shows the removal rate of TNZ and the corresponding first-order kinetic constants for different photocatalyst samples under the condition of VLIN. The removal rate of TNZ by different photocatalysts followed the order ZS > ZnBiGdO_4_ > SnS_2_ > N-T. The removal rate of TNZ by using ZS was increased by 1.12 times, 1.26 times, or 2.41 times compared with that by using ZnBiGdO_4_, SnS_2_, or N-T. In addition, the kinetic constant which derived from the effect of TNZ concentration and VLIN time by using ZS was increased by 2.55 times, 3.45 times, or 10.76 times compared with that by using ZnBiGdO_4_, SnS_2_, or N-T, respectively. Figure S7a shows the curves of the quintic experimental cycles for degrading TNZ with ZS as a photocatalyst under the condition of VLIN. Figure S7b displays the kinetic curves of five experimental cycles for degrading TNZ by using ZS under the condition of VLIN. Figure S7c indicates the removal efficiencies and kinetic constants of five cyclical tests for degrading TNZ with ZS as a photocatalyst under the condition of VLIN. Figure S7d illustrates the quintic experimental cycle curves of TOC concentration change during the process of degrading TNZ with ZS as a photocatalyst under the condition of VLIN. Figure S7e shows the kinetic curves of five cyclical tests for removing the TOC concentration by using ZS under the condition of VLIN. Figure S7f illustrates the mineralization efficiencies and kinetic constants of five cyclical tests for removing the TOC concentration with ZS as a catalyst under the condition of VLIN. After quintic experimental cycles, the removal rate of TNZ by using ZS still remained at 95.9%. Figure S8a,b show the XRD patterns and the XPS spectra of the unused ZS and the used ZS. The additional peaks were not observed in the images of the used ZS after five cycle experiments. Above results indicated that the ZS catalyst possessed extremely strong stability in photocatalytic activity.

Figure 5d shows the variation curves of *TOC* concentration during the process of removing TNZ in wastewater using different photocatalysts under VLIN. The prepared catalysts achieved significant mineralization efficiency for removing *TOC* concentration, namely, the mineralization results of the *TOC* concentration were consistent with the TNZ degradation results. Figure 5e shows the plots derived from the effect of ln(*TOC*_0_/*TOC_t_*) on irradiation time of visible light; as a result, the plots fitted a first-order kinetic model, which helped to calculate the first-order kinetic constant during the mineralization process of *TOC* concentration. The first-order kinetic constant that originated from the effect of ln(*TOC*_0_/*TOC_t_*) on irradiation time of visible light was calculated using the standard Formula $\ln(TOC_{0}/TOC_{t})=k_{TOC}t$, where *TOC_t_* represents the total organic carbon concentration at the intermediate state, and *TOC*_0_ represents the total organic carbon concentration at the initial state. In order to more clearly demonstrate the mineralization performance of *TOC* concentration, Figure 5f shows the mineralization efficiency of *TOC* concentration and kinetic constants by using the prepared photocatalysts. The mineralization rates of *TOC* concentration by using the different photocatalysts were in the following order: the mineralization rate of *TOC* concentration by using ZS > the mineralization rate of *TOC* concentration by using ZnBiGdO_4_ > the mineralization rate of *TOC* concentration by using SnS_2_ > the mineralization rate of *TOC* concentration by using N-T. It is worth noting that ZS exhibited the most excellent mineralization performance for varying the *TOC* concentration during the process of degrading TNZ. The mineralization efficiency for removing *TOC* concentration with ZS as a catalyst was 1.15 times higher than that with ZnBiGdO_4_ as a catalyst, 1.28 times higher than that with SnS_2_ as a catalyst, and 2.57 times higher than that with N-T as a catalyst. Meanwhile, the kinetic constant *k_TOC_* that was gained from the dynamical curve between *TOC* concentration and VLIN time by using ZS as catalyst increased by approximately 2.26 times, 2.98 times, or 9.44 times compared with that by using ZnBiGdO_4_, SnS_2_, or N-T as a catalyst. In accordance with Figure S7d–f, after quintic experimental cycles, the removal rate of TOC concentration by using ZS as a catalyst remained at 94.51%. The above results indicated that the prepared ZS photocatalyst possessed extremely strong stability in photocatalytic activity.

**Figure 6:**
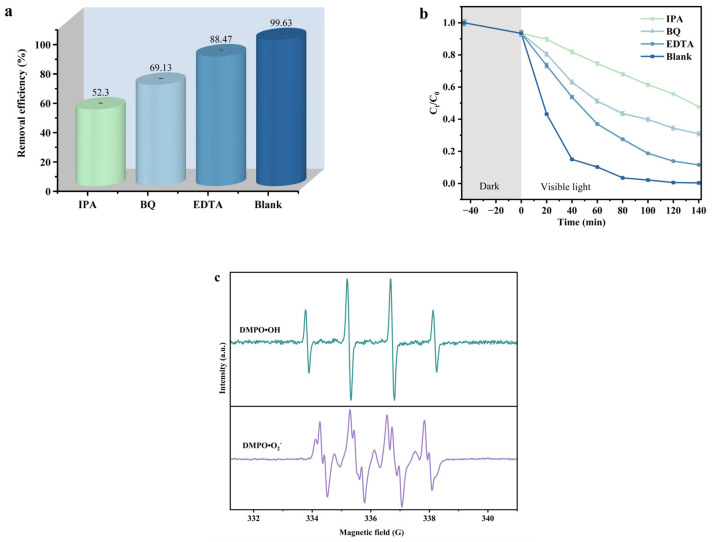
Impact of different radical scavengers on (**a**) TNZ saturation concentration, (**b**) removal efficiency of TNZ and (**c**) EPR spectrum for DMPO·O^2−^ and DMPO·OH with ZS as photocatalyst.

In order to investigate the contribution of active species that derived from ZS in the photocatalytic degradation of TNZ, we conducted systematic free radical trapping experiments. The roles of various active species were evaluated by adding different free radical scavengers. Isopropyl alcohol (IPA) was used to capture hydroxyl radicals (•OH), meanwhile, benzoquinone (BQ) was utilized to capture superoxide anions (•O_2_^−^), and ethylenediaminetetraacetic acid (EDTA) was used to capture photoinduced holes (h^+^). The above experimental results are shown in Figure 6a,b. Without the addition of IPA, BQ, and EDTA, the removal rate of TNZ was 99.63%. However, the removal rate of TNZ was decreased to 52.30%, 69.13%, or 88.47% when IPA, BQ, or EDTA was utilized as scavenger, respectively. The above results indicated that the contribution of hydroxyl radicals (•OH) was significantly higher than that of superoxide anions (•O_2_^−^) and that of photoinduced holes (h^+^) during the photocatalytic degradation of TNZ with ZS as a catalyst.

The electron paramagnetic resonance (EPR) experiments were realized by using DMPO (5,5-dimethy-l-pyrroline N-oxide) as a trapping agent. Figure 6c displays the EPR spectrum for DMPO·O^2−^ and DMPO·OH. According to Figure 6c, the reactive species hydroxyl radical (•OH) was produced and detected in during the photocatalytic degradation of TNZ with ZS as a catalyst. From Figure 6c, it could be observed that the ratio of the four peaks for the hydroxyl radical was 1:2:2:1, which was a unique signal that belonged to the hydroxyl radical. When dimethyl sulfoxide was used as a dispersant and DMPO was utilized as a trapping agent, the reactive species superoxide anion (•O^2−^) that was produced during the photocatalytic degradation of TNZ with ZS as catalyst was detected. The concentration of •OH was significantly higher than that of •O^2−^, and simultaneously, the above result was consistent with the results that were obtained from free radical scavenging experiments.

#### 2.2.2. The Possible Photocatalytic Degradation Mechanism of ZS

**Figure 7:**
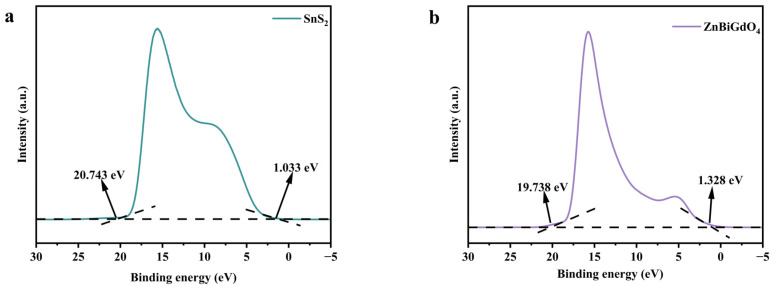
UPS spectra of (**a**) SnS_2_ and (**b**) ZnBiGdO_4_.

In order to obtain information about valence electron interactions near the Fermi level of the catalyst and valence band (or occupied states) information, ultraviolet photoelectron spectroscopy experiments were performed on the catalytic samples. The experimental results are shown in Figure 7a,b. The ionization potential of the valence band for SnS_2_ was 1.490 eV, and the ionization potential of the valence band for ZnBiGdO_4_ was 2.790 eV. The electrochemical potential of the conduction band for SnS_2_ or ZnBiGdO_4_ was calculated according to the following Formula.

(5) $$E_{VB}=E_{g}+E_{CB}$$

The electrochemical potential of the conduction band for SnS_2_ was −0.585 eV, and concurrently, the electrochemical potential of the conduction band for ZnBiGdO_4_ was 0.953 eV.

The electrochemical potential of the conduction band for SnS_2_ was more negative than the standard potential of O_2_/•O^2−^ (−0.33 eV vs. NHE), indicating that SnS_2_ possessed a stronger reducing ability. The above result meant that the electrons located in the conduction band of SnS_2_ were captured by the adsorbed oxygen molecules, and as a result, oxygen molecules could be easily reduced for generating superoxide anions (•O^2−^). The ionization potential of the valence band for ZnBiGdO_4_ was more positive than the standard potential of OH^−^/•OH (2.38 eV vs. NHE), indicating that ZnBiGdO_4_ had a stronger oxidizing ability [38,39]. The above result meant that the photoinduced holes located in the valence band of ZnBiGdO_4_ could oxidize water or hydroxide ion (hydroxyl) for generating the hydroxyl radicals (•OH). The above result analysis was consistent with the experimental results that were obtained by EPR analysis.

**Figure 8:**
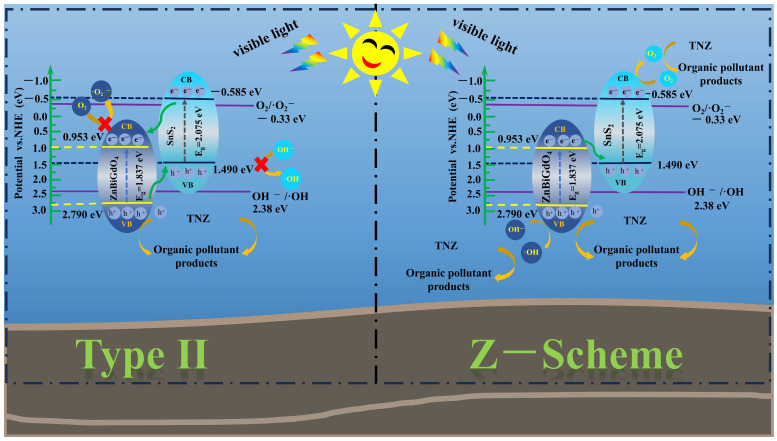
Plausible photodegradation mechanism of TNZ with ZS as photocatalyst.

Based on the exhaustive analysis of previous experimental results, the internal charge transfer path of ZS was plotted. According to the band structure of ZnBiGdO_4_ or SnS_2_, Figure 8 shows two degradation mechanisms of TNZ, direct Z-scheme and Type II [40]. As to the degradation mechanism of TNZ for Type II, more photoinduced electrons converged on the conduction band (CB) of ZnBiGdO_4_, and concurrently, more photoinduced holes converged on the valence band (VB) of SnS_2_. However, because the ionization potential of the conduction band for ZnBiGdO_4_ was more positive than the standard potential of O_2_/•O^2−^ (−0.33 eV vs. NHE), the •O^2−^ could not be generated. Meanwhile, the ionization potential of the valence band for SnS_2_ was smaller than the standard potential of OH^−^/•OH (2.38 eV vs. NHE); thus, the •OH could not be generated. Such a reaction mechanism was inconsistent with the above free radical scavenging experiments and electron paramagnetic resonance experiments.

In regard to the degradation mechanism of TNZ for the direct Z-scheme, the photoinduced electrons moved from the CB of ZnBiGdO_4_ to the VB of SnS_2_ and recombined with the photoinduced holes when ZS was irradiated by visible light. The direct Z-scheme mechanism not only promoted the efficient separation of the photoinduced carriers but also ensured the simultaneous existence of raised reductive potential and elevated oxidative potential. Under such conditions, the photoinduced holes which located in the valence band of ZnBiGdO_4_ could oxidize water or hydroxide ions (hydroxyl) for producing the hydroxyl radical (•OH); simultaneously, the photoinduced electrons which located in the conduction band of SnS_2_ reduced oxygen molecules for generating the superoxide anion (•O^2−^). Above analytical results were consistent with the experimental results which were obtained from the free radical scavenging experiments and the resultant analysis of EPR. Above results meant that the degradation mechanism of TNZ with ZS as a catalyst could be the same as the direct Z-scheme heterojunction degradation mechanism [41].

#### 2.2.3. Possible Degradation Pathways of TNZ

**Figure 9:**
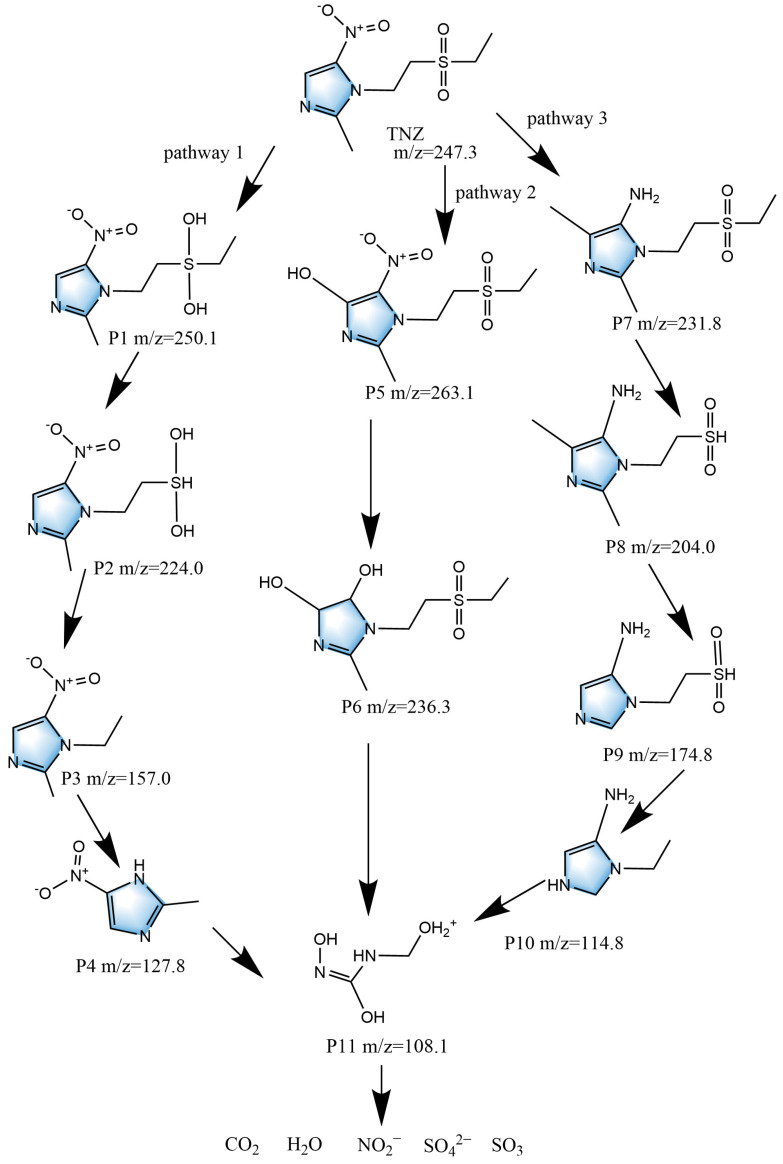
Viable photodegradation pathways for TNZ under VLIN with ZS heterojunction as catalyst.

Based on the existing literature reports and our liquid chromatography-mass spectrometry (LC-MS) detected results, this study speculated the possible degradation pathways of TNZ (*m*/*z* = 247.3) and the related intermediate products of TNZ. As shown in Figure 9, three different degradation pathways of TNZ were analyzed.

In the first degradation pathway of TNZ, the degradation process of TNZ might start with the reduction reaction of the sulfonyl group in the sulfone; as a result, the intermediate product P1 (*m*/*z* = 250.1) was formed [42,43,44]. Subsequently, the ethyl group that was connected to the S atom was removed to form P2 (*m*/*z* = 224.0) [42,43,44]. The cleavage of the C–S bond formed P3 (*m*/*z* = 157.0) [42,43], which then underwent dealkylation for forming a compound that was named as 2-methyl-5-nitroimidazole, P4 (*m*/*z* = 127.8) [42,43,44]. The imidazole ring in P4 was attacked by active molecules; as a result, the above imidazole ring in P4 was decomposed for forming P11 (*m*/*z* = 108.1) [42,43,44,45].

In the second degradation pathway of TNZ, the degradation process of TNZ might begin with the hydroxylation of the imidazole ring; as a consequence, the formation of the intermediate product P5 (*m*/*z* = 263.1) and the intermediate product P6 (*m*/*z* = 236.3) [46,47] was realized. Subsequently, the ring opening of the N-heterocycle and the loss of a methyl group might ultimately lead to the formation of P11 (*m*/*z* = 108.1) [45].

In the third degradation pathway of TNZ, P7 (*m*/*z* = 231.8) was first generated by means of a cyclization reaction; during the process of the above cyclization reaction, the nitro group in the TNZ structure was converted to an amino group, and a methyl group was added to the imidazole ring [42]. Subsequently, the removal of the ethyl group in P7 formed P8 (*m*/*z* = 204.0) [42]. The removal of the methyl group on the imidazole ring in P8 formed P9 (*m*/*z* = 174.8) [42]. The removal of the sulfonyl group in P9 formed the small molecule P10 (*m*/*z* = 114.8) [42]. Finally, P11 (*m*/*z* = 108.1) was formed by means of an imidazole ring-opening reaction [45].

Eventually, P11 (*m*/*z* = 108.1) was further degraded into carbon dioxide (CO_2_), water (H_2_O), sulfur trioxide (SO_3_), and inorganic anions which included sulfate (SO_4_^2−^) and nitrite (NO_2_^−^).

## 3. Experiment and Methods

### 3.1. Materials and Reagents

Aladdin Group Chemical Reagent Co., Ltd. in Shanghai, China was the supplier of ZnCl_2_ (purity = 99.995%), BiCl_3_ (purity = 99.999%), GdCl_3_ (purity = 99.99%), Isopropanol (IPA, C_3_H_8_O, purity ≥ 99.999%), and TNZ (C_8_H_13_N_3_O_4_S, purity ≥ 98%). S element simple substance powder was purchased from Sinopharm Group Chemical Reagent Co., Ltd. in Shanghai, China. Ethylenediaminetetraacetic acid (EDTA, C_10_H_16_N_2_O_8_, purity = 99.995%) was purchased from Merck Group Chemical Reagent Co., Ltd. in Shanghai, China. Benzoquinone (BQ, C_6_H_4_O_2_, purity ≥ 99.5%) and SnCl_2_·2H_2_O (purity = 99.99%) were purchased from Macklin Biochemical Co., Ltd. in Shanghai, China. All chemical reagents were used as received reagents without further purification.

### 3.2. Preparation of ZnBiGdO_4_

The ZnBiGdO_4_ photocatalyst was prepared using the co-precipitation method. First, the same molar amounts of ZnCl_2_, BiCl_3_, and GdCl_3_ were weighed and dissolved in 800 mL of water or inorganic acid (hydrochloric acid). The resulting solution was magnetically mixed for 180 min, and then slowly poured into NaOH solution under high-speed stirring condition to form a suspension. The suspension was immersed in a boiling water bath. The time could range from 90 min to 210 min. Then the resulting suspension was washed 20 times to 25 times with deionized water.

Subsequently, it was allowed to stand for 2 h and filtered. The filtered product was dried at 116 °C for 210 min, and the powder that was obtained after drying was dried in an air oven at 460 °C for 4 h; then, the above drying powder was ground for obtaining ZnBiGdO_4_ single crystal powder [48]. In reference [48], the catalyst has been prepared via the co-precipitation method and a facile sol–gel method. In this study, the ZnBiGdO_4_ photocatalyst was also prepared using the co-precipitation method. The ZnBiGdO_4_ sample was prepared in duplicate by using the same method for demonstrating reliability and reproducibility of synthesis.

### 3.3. Synthesis of SnS_2_

SnS_2_ powder photocatalyst was synthetized using a low-temperature melting method. S powder and SnCl_2_·2H_2_O were mixed at a molar ratio of 5:2 and ground in an agate mortar for 20 min. Subsequently, the mixture was transferred to a crucible and covered with a lid. The crucible containing the mixture was placed in a thermostatic electric oven and heated at 125 °C (the lowest temperature for preparing pure-phase SnS_2_ via the current melting method) for 6 h. The product was allowed to cool naturally at room temperature for 5 h; then, it was washed with carbon disulfide (CS_2_), deionized water, and ethanol. The above product was then dried in a vacuum oven at 90 °C for 12 h and stored in a desiccator for later use. The pure-phase SnS_2_ photocatalyst was successfully prepared [49]. In reference [49], a molten method with the characteristics of low temperature, air environment, simplicity, cost-effectiveness, and easy scale-up was tried for the synthesis of the highly photocatalytical active SnS_2_. In this study, the SnS_2_ powder photocatalyst was synthesized by using a low-temperature melting method. The SnS_2_ powder photocatalyst was synthetized by the same method twice. Figure S1 shows the XRD pattern of SnS_2_ which was synthetized firstly (SnS_2_-1) and the XRD pattern of SnS_2_ which was synthetized secondly (SnS_2_-2).

### 3.4. Production Process of ZS

The ZS photocatalyst was prepared using the microwave-assisted solvothermal technique. The ZnBiGdO_4_ powder, which was prepared by the co-precipitation method, and the SnS_2_ powder, which was prepared by the low-temperature melting method, were mixed at a molar ratio of 2:5 with octanol and sonicated for 2 h. The mixture was transferred to a quartz bottle and heated in a microwave synthesizer (CEM Discover) at 180 °C for 2 h. After cooling, the product was separated using a magnet, washed with deionized water and ethanol, and dried at 80 °C for 12 h. Furthermore, the ZS photocatalyst was successfully prepared [50,51,52]. In reference [50], the photocatalysts were prepared by using a special two-step microwave-assisted solvothermal technique. In reference [51], the novel heterojunction photocatalysts were prepared by using a fast and stable microwave-assisted method and characterized by X-ray diffractometry, X-ray photoelectron spectroscopy, scanning electron microscopy, ultraviolet-visible spectroscopy, and fluorescence spectroscopy. In reference [52], a novel direct Z-scheme heterojunction photocatalyst was synthesized by using a facile and rapid microwave heating method. In this study, the ZS heterojunction photocatalyst was prepared by using the microwave-assisted solvothermal technique. In the preparation method of the ZS photocatalyst described in this paper, some contents derived from the references [19,20,21] were adopted and the further innovation method was created based on them. The ZS photocatalyst was prepared twice by using the same technique and the reproducibility of the ZS photocatalyst was circumstantiated. Figure S2 shows the XRD pattern of the ZS photocatalyst which was prepared firstly (ZS-1) and the XRD pattern of the ZS photocatalyst which was prepared secondly (ZS-2). According to Figure S2, the ZS photocatalyst possessed reproducibility and reliability by using the same preparation method.

### 3.5. Method for Synthesizing N-Doped TiO_2_

The TiO_2_ powder was placed on a platinum plate and then transferred to a tube furnace. A mixed gas of NH_3_/Ar (volume ratio of 3:2) was introduced into the tube at a flow rate of 50 mL/min and maintained for 2 h to remove air and moisture. When the gas flow rate was stabilized to 200 mL/min, the furnace temperature was raised from indoor temperature to 600 °C at a rate of 10 °C/min. Subsequently, the temperature was maintained at 600 °C for 5 h with the introduction of NH_3_/Ar mixed gas (volume ratio of 3:2). The furnace temperature was cooled to room temperature at a rate of 20 °C/min under the same gas flow rate. Subsequently, the sample was taken out, and the obtained sample was ground into powder; ultimately, the N-doped TiO_2_ (N-T) sample was successfully preparing [53]. In reference [53], TiO_2_ doped with various loadings of nitrogen was prepared by nitridation of a nano-TiO_2_ powder in an ammonia/argon atmosphere at a range of temperatures from 400 °C to 1100 °C. Similarly, in the N-doped TiO_2_ photocatalyst synthesis process described in this paper, a mixed gas of NH_3_/Ar passed into TiO_2_. The sample preparation methods in this study retained the advantages of the previously reported approaches and incorporated further innovations. Moreover, the method of preparing the N-T sample was repeated to give evidence of reproducibility.

### 3.6. Photoelectrochemistry Experiment

The photoelectrochemical performance was measured using a CHI-660D electrochemical workstation (Chenhua Instruments Co., Ltd., Shanghai, China). The workstation was equipped with three electrodes: platinum as the counter electrode, silver/silver chloride as the reference electrode, and the synthesized catalyst-coated conductive substrate as the working electrode. The electrolyte was a 0.3 mol/L sodium sulfate solution. A total of 2.5 mg of photocatalyst powder was weighed and dispersed in 250 μL of ethanol-ethylene glycol mixed solvent (volume ratio of 1:1), followed by sonication for 40 min and subsequent magnetic stirring for 3 h to obtain a homogeneous suspension. Then, 30 μL of above suspension was drop-coated onto the surface of a pretreated glassy carbon electrode (approximately 0.3 mg/cm^2^, assuming a glassy carbon electrode area of 0.07 cm^2^). The working electrode was prepared by drying at room temperature for 30 min. In order to systematically investigate the electrochemical properties of the photocatalysts, electrochemical impedance spectroscopy (EIS) tests were also performed in KCl solution. All tests were performed at room temperature.

### 3.7. Experimental Setup and Procedure

The degradation performance of three different catalysts (ZnBiGdO_4_, SnS_2_, and ZS) on TNZ was evaluated using a CEL-LB70 photocatalytic reaction system (CEL-LB70, Zhongjiao Jinyuan Technology Co., Ltd., Beijing, China). First, 0.2 g photocatalytic powder (ZnBiGdO_4_, SnS_2_, or ZS) was added to the 300 mL TNZ solution at a concentration of 0.03 mmol/L. Before the formal photocatalytic reaction, the system was first pretreated with dark adsorption for 45 min under dark conditions. This process ensured that the catalyst particles were evenly dispersed in the reaction solution and promoted the TNZ molecules to reach the adsorption–desorption equilibrium state on the catalyst surface. More importantly, the above process could accurately distinguish and exclude the contribution of physical adsorption to the degradation efficiency. Thereby, the subsequent photocatalytic degradation data reflected the photocatalytic activity of the photocatalysts.

The photocatalytic degradation reaction was performed under VLIN using a 500 W xenon lamp (equipped with a 420 nm filter) as the light source. During the reaction, 5 mL of reaction solution was collected every 20 min. After centrifugation at 7200 rpm for 10 min, the supernatant was taken out for further analysis using an Agilent 200 high-performance liquid chromatography system (Agilent Technologies, Palo Alto, CA, USA).

In order to systematically investigate the mineralization behavior of total organic carbon (TOC) during the degradation of TNZ, a TOC analyzer (TOC-5000 A, Shimadzu Corporation, Kyoto, Japan) was used to quantitatively analyze the degree of mineralization of the reaction system. During the experiment, the potassium biphthalate (KHC_8_H_4_O_4_) standard aqueous solution was used as the calibration standard substance. A standard curve was established within the carbon concentration range of 0 mg/L–100 mg/L.

In addition, in order to deeply analyze the intermediate products of the degradation process, the Thermo Quest LCQ Duo liquid chromatography-mass spectrometry (LC-MS) system (Thermo Fisher Scientific Corporation, Waltham, MA, USA) was used for product identification analysis. After the photocatalytic reaction was completed, 20 μL of the sample was automatically extracted from the reaction solution and injected into the LC-MS system. The system was equipped with a Beta Basic-C18 chromatographic column, with methanol-deionized water (60:40) as the mobile phase, and the flow rate was kept constant at 0.2 mL/min. The mass spectrometry was set to detect a mass-to-charge ratio (*m*/*z*) of 50 to 500. Subsequently, daughter ion fragments were searched to elucidate the possible degradation pathways and intermediate products of FNT.

### 3.8. Characterization

Crystallographic data could be obtained using a Shimadzu XRD-6000 diffractometer (Kyoto, Japan). Fourier transform infrared spectroscopy (FTIR) analysis was performed using a WQF-530A spectrometer from Beifen-Ruili Analytical Instrument (Group) Co., Ltd. in Beijing, China for revealing the information such as the molecular structure, chemical bond types, and functional group composition of the photocatalyst. Raman spectroscopy analysis was performed using an INVIA0919-06 system provided by RENSHAW plx, based in Wotton-under-Edge, Gloucestershire, UK, for analyzing the chemical structure, lattice dynamics, and molecular interactions of the photocatalyst. The surface morphology and lattice fringes of the photocatalysts were analyzed using a transmission electron microscope (TEM) (Talos F200X G2 instrument from Thermo Fisher Scientific, located in Waltham, MA, USA); concurrently, the EDS energy spectrum analysis was realized to determine the elemental composition and content of the photocatalysts. X-ray photoelectron spectroscopy (XPS) (PHI 5000 VersaProbe instrument from ULVAC-PHI in Maoqi City, Japan) was performed to analyze the surface elemental composition and chemical states of the photocatalysts. The optical properties of the samples were evaluated using a UV-Vis diffuse reflectance spectrophotometer (UV-Vis DRS). Additionally, the photoluminescence spectra (PL) and the fluorescence lifetime of the photocatalysts were tested by a FLS980 spectrophotometer from Edinburgh Instruments Ltd. in Edinburgh, UK. The excitation wavelength of the sample was 300 nm in the PL experiment. Finally, the active free radicals in the samples were detected by electron paramagnetic resonance (EPR) spectroscopy using an A300 instrument from Bruker Corporation in Karlsruhe, Germany.

In addition, an Escalab 250 xi instrument from Thermo Fisher Scientific in Waltham, MA, USA was used to measure the ionization potential of the valence band of the photocatalyst by ultraviolet photoelectron spectroscopy (UPS).

## 4. Conclusions

In summary, the direct Z-scheme heterojunction photocatalyst ZS was successfully synthesized for the first time with the microwave-assisted solvothermal method. ZnBiGdO_4_ belonged to the body-centered tetragonal system with a spinel structure, and space group was I41/AMD; concurrently, the cell parameters for ZnBiGdO_4_ were a = b = 14.622367 Å, c = 9.513909 Å. The band gap width of SnS_2_, ZnBiGdO_4_, and the ZS heterojunction photocatalyst was found to be 2.075 eV, 1.837 eV, and 1.821 eV. Each sample was prepared in duplicate by using the same method. The experimental results indicated that there was a significant Z-scheme charge transfer mechanism between ZnBiGdO_4_ and SnS_2_; as a result, the ZS heterojunction photocatalyst not only significantly improved the separation efficiency of the photoinduced carriers, but also maintained strong oxidative ability and strong reductive ability. In the experiment of degrading TNZ, the ZS heterojunction photocatalyst exhibited an excellent photocatalytic performance. The removal rate of TNZ with the ZS heterojunction as a catalyst was 1.12 times, 1.26 times, and 2.41 times that with ZnBiGdO_4_, SnS_2_, and N-T as a catalyst. The mineralization efficiency for removing *TOC* concentration by using ZS was 1.15 times, 1.28 times, or 2.57 times higher compared with that by using ZnBiGdO_4_, SnS_2_, or N-T. By means of free radical trapping experiments and electron paramagnetic resonance experiments, the key oxydic role of the active species for ·OH, ·O_2_^−^, and h^+^ during the degradation process of TNZ was confirmed as having the following order: ·OH > ·O_2_^−^ > h^+^. The XRD patterns and the XPS spectra of the unused ZS and the used ZS did not change, indicating that the ZS catalyst possessed extremely strong stability for the photocatalytic activity of TNZ. The ZS photocatalyst that was prepared for the first time displayed the same XRD pattern compared with the ZS photocatalyst that was prepared for the second time, indicating that the ZS catalyst possessed reproducibility and reliability by using the same preparation method. Simultaneously, the ZS sample maintained good stability and reliability even after five cycle experiments. In addition, this study also systematically elucidated the photocatalytic degradation mechanism and reaction pathway of TNZ. These findings suggest that the ZS heterojunction photocatalyst has broad application prospects in the photocatalytic degradation of antibiotic organic pollutants that derive from pharmaceutic wastewater.

## Data Availability

Data are contained within the article.

## Supplementary Materials

The following supporting information can be downloaded at: https://www.mdpi.com/article/10.3390/ijms26178366/s1.
