# Supplementary material for: Innovative Z-Scheme Heterojunction Photocatalyst ZnBiGdO4/SnS2 for Photocatalytic Degradation of Tinidazole Under Visible Light Irradiation
Source: Int J Mol Sci. 2025 Aug 28;26(17):8366. doi: 10.3390/ijms26178366 (PMC12429297; doi:10.3390/ijms26178366)
Supplement: Supplementary file 1 [file ijms-26-08366-s001.zip › ijms-3797465-supplementary.pdf]

Innovative Z-scheme Heterojunction Photocatalyst $ZnBiGdO_4/SnS_2$ for Photocatalytic Degradation of Tinidazole under Visible Light Irradiation

Jingfei Luan [1,2,*], Boyang Liu [1], Liang Hao [1], Wenchen Han [1] and Anan Liu [1]

1 School of physics, Changchun Normal University, Changchun 130032, China; boyangliu152@outlook.com (B. L.); 19845486007@139.com (L.H.); han18635869581@outlook.com (W.H.); AnanLiu2001@outlook.com (A.L.)
2 State Key Laboratory of Pollution Control and Resource Reuse, School of the Environment, Nanjing University, Nanjing 210093, China
* Correspondence: jfluan@nju.edu.cn; Tel.: +86-199-5193-9498

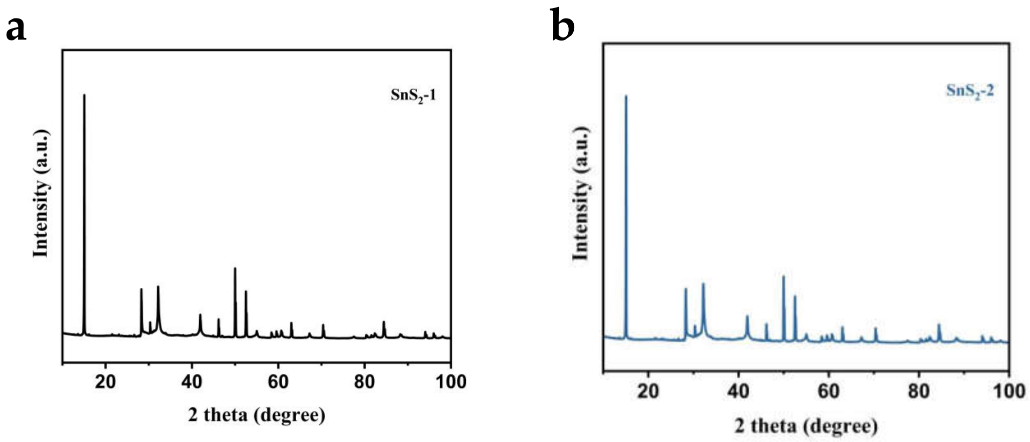

Figure S1. (**a**) The XRD patterns of SnS_2 which was synthetized firstly and (**b**) the XRD pattern of SnS_2 which was synthetized secondly by the low-temperature melting method.

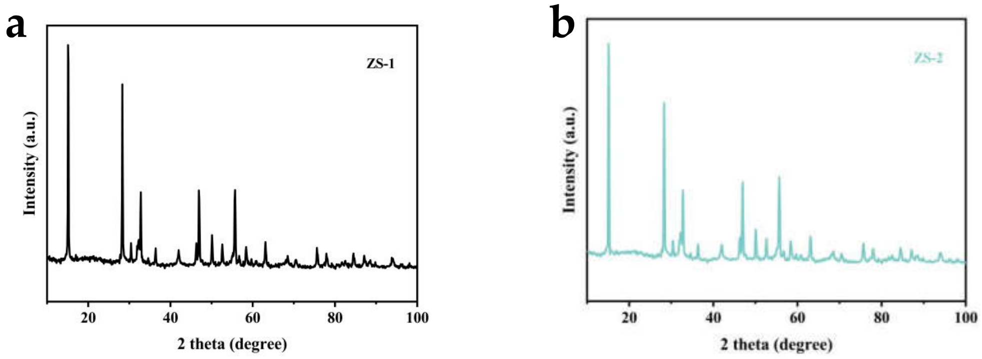

Figure S2. (**a**) The XRD patterns of ZS which was prepared firstly and (**b**) the XRD pattern of ZS which was prepared secondly using the microwave-assisted solvothermal technique.

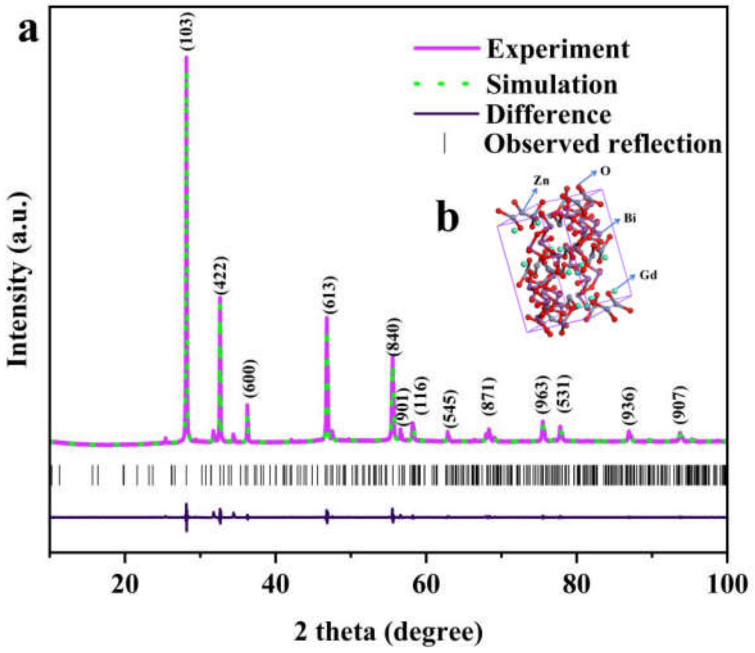

Figure S3. (**a**) The XRD pattern and the Pawley refinement result and (**b**) the atomic architecture of $ZnBiGdO_4$.

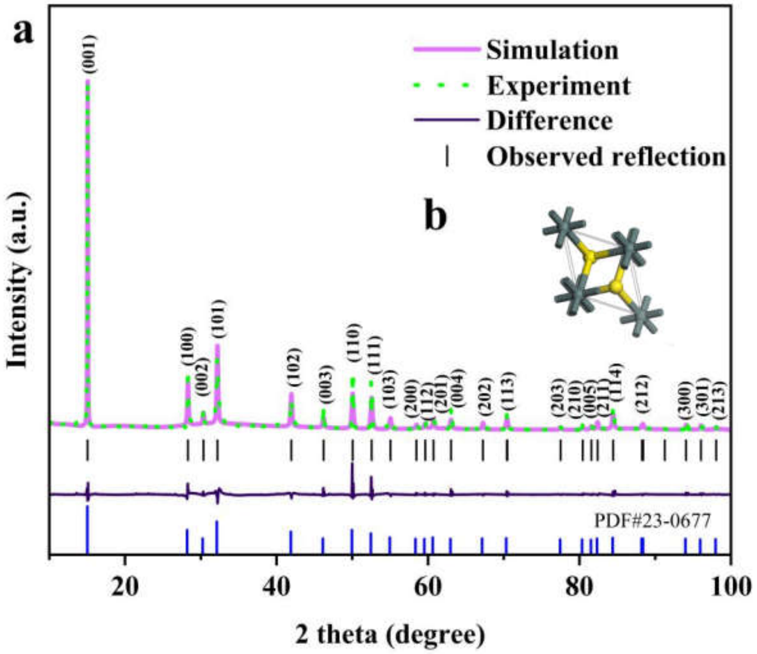

Figure S4. (**a**) The XRD pattern and the Pawley refinement result and (**b**) the atomic architecture of SnS_2.

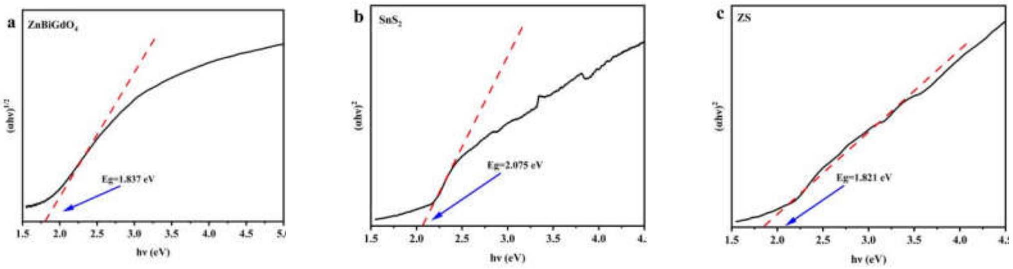

Figure S5. (a) Corresponding plot of $(\alpha h\nu)^{1/2}$ and $h\nu$ for $ZnBiGdO_4$; (**b**) corresponding plot of $(\alpha h\nu)^2$ and $h\nu$ for SnS_2 and (**c**) corresponding plot of $(\alpha h\nu)^2$ and $h\nu$ for ZS.

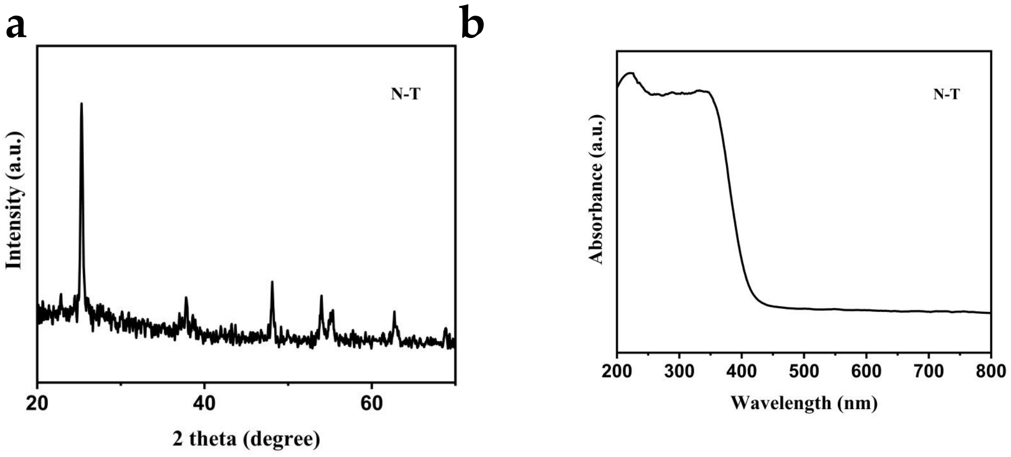

Figure S6. (a) The XRD spectrum of the N-doped TiO_2; **(b)** the UV-Vis diffuse reflectance spectra of the N-doped TiO_2.

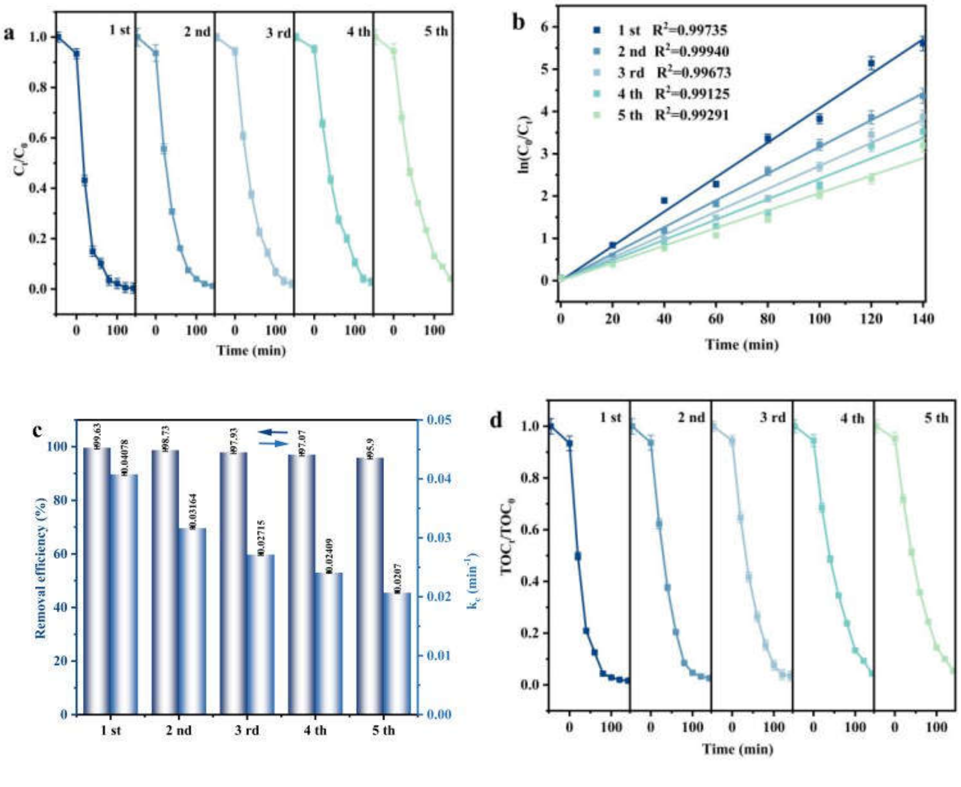

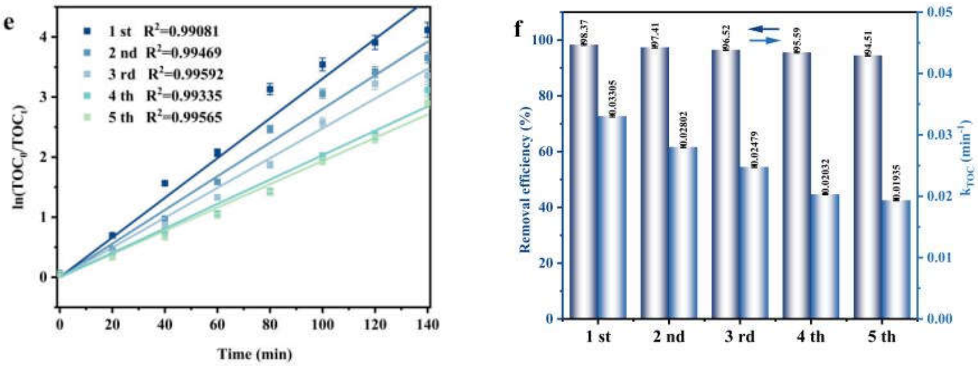

Figure S7. (a) Photodegradation; **(b)** kinetic curves, and **(c)** removal efficiencies and kinetic constants for five cyclical tests for degrading TNZ; **(d)** mineralization; **(e)** kinetic curves, and **(f)** mineralization efficiencies and kinetic constants of five cyclical tests for removing TOC concentration with ZS as catalyst.

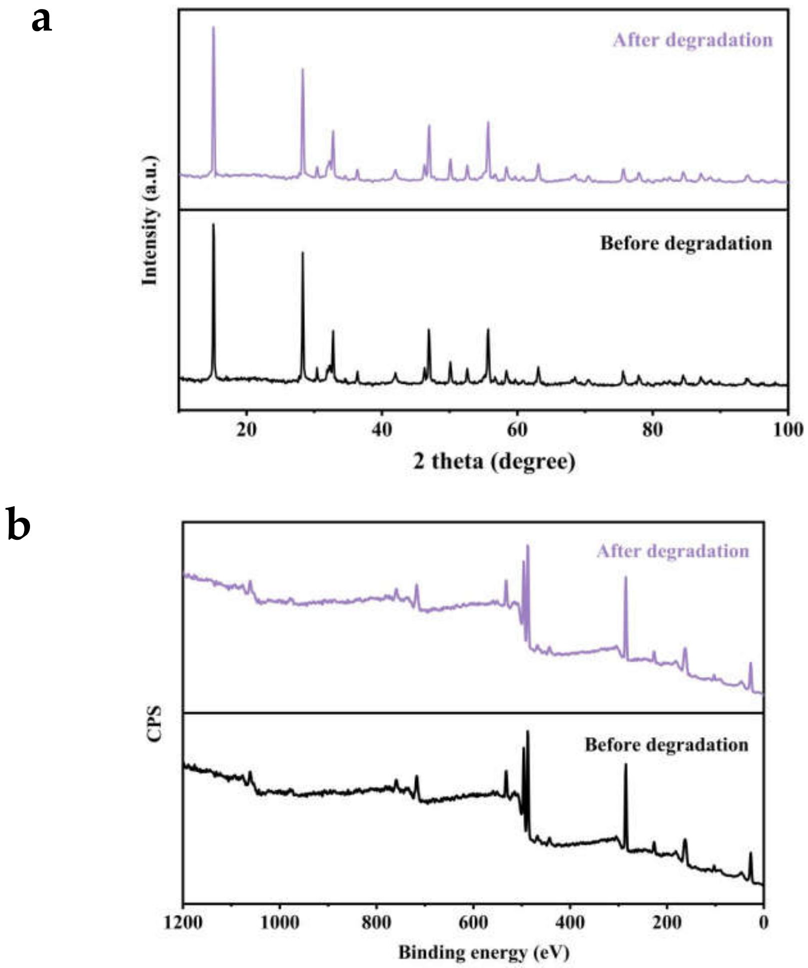

Figure S8. (a) The XRD patterns of the unused ZS and the used ZS; (b) The XPS patterns of the unused ZS and the used ZS.

Table S1. Configurable properties of $ZnBiGdO_4$ which was fabricated using the co-precipitation method.

**Atom**	x	y	z	**Occupation Factor**
Zn	0.39003	0.00000	0.00000	1
Bi	0.00000	0.11132	0.22252	1
Gd	0.15752	0.09252	0.87500	1
O(1)	0.00000	0.97728	0.30179	1
O(2)	0.15267	0.05286	0.12528	1
O(3)	0.39217	0.14216	0.12500	1

Table S2. Configurable properties of SnS_2 which was fabricated using the low-temperature melting method.

**Atom**	x	y	z	**Occupation Factor**
S	0.670	0.330	-0.259	1
Sn	0	0	0	1
